# Buccal mucosa micronuclei counts in relation to exposure to low dose-rate radiation from the Chornobyl nuclear accident and other medical and occupational radiation exposures

**DOI:** 10.1186/s12940-017-0273-x

**Published:** 2017-06-23

**Authors:** D. Bazyka, S. C. Finch, I. M. Ilienko, O. Lyaskivska, I. Dyagil, N. Trotsiuk, N. Gudzenko, V. V. Chumak, K. M. Walsh, J. Wiemels, M. P. Little, L.B. Zablotska

**Affiliations:** 1 National Research Center for Radiation Medicine, 53 Melnikov Street, Kyiv, 04050 Ukraine; 2 Rutgers-Robert Wood Johnson Medical School, 5635, 675 Hoes Lane W, Piscataway Township, New Brunswick, NJ 08854 USA; 3 UCSF Box 0520, Division of Neuroepidemiology, Helen Diller Family Comprehensive Cancer Center, University of California San Francisco, 505 Parnassus Avenue, San Francisco, CA 94143-0520 USA; 4 Box 0520, Laboratory of Molecular Epidemiology, University of California San Francisco Comprehensive Cancer Center, 1450 3rd Street, San Francisco, CA 94158 USA; 5 Division of Cancer Epidemiology and Genetics, National Cancer Institute, National Institutes of Health, Department of Health and Human Services, Radiation Epidemiology Branch, Room 7E546, 9609 Medical Center Drive, Bethesda, MD 20892-9778 USA; 6 Department of Epidemiology and Biostatistics, School of Medicine, University of California, San Francisco, 3333 California St, San Francisco, CA 94118 USA

**Keywords:** Micronucleus, Ionizing radiation, Chornobyl, Chromosome aberrations, Radiography

## Abstract

**Background:**

Ionizing radiation is a well-known carcinogen. Chromosome aberrations, and in particular micronuclei represent an early biological predictor of cancer risk. There are well-documented associations of micronuclei with ionizing radiation dose in some radiation-exposed groups, although not all. That associations are not seen in all radiation-exposed groups may be because cells with micronuclei will not generally pass through mitosis, so that radiation-induced micronuclei decay, generally within a few years after exposure.

**Methods:**

Buccal samples from a group of 111 male workers in Ukraine exposed to ionizing radiation during the cleanup activities at the Chornobyl nuclear power plant were studied. Samples were taken between 12 and 18 years after their last radiation exposure from the Chornobyl cleanup. The frequency of binucleated micronuclei was analyzed in relation to estimated bone marrow dose from the cleanup activities along with a number of environmental/occupational risk factors using Poisson regression adjusted for overdispersion.

**Results:**

Among the 105 persons without a previous cancer diagnosis, the mean Chornobyl-related dose was 59.5 mSv (range 0–748.4 mSv). There was a borderline significant increase in micronuclei frequency among those reporting work as an industrial radiographer compared with all others, with a relative risk of 6.19 (95% CI 0.90, 31.08, 2-sided *p* = 0.0729), although this was based on a single person. There was a borderline significant positive radiation dose response for micronuclei frequency with increase in micronuclei per 1000 scored cells per Gy of 3.03 (95% CI -0.78, 7.65, 2-sided *p* = 0.1170), and a borderline significant reduction of excess relative MN prevalence with increasing time since last exposure (*p* = 0.0949). There was a significant (*p* = 0.0388) reduction in MN prevalence associated with bone X-ray exposure, but no significant trend (*p* = 0.3845) of MN prevalence with numbers of bone X-ray procedures.

**Conclusions:**

There are indications of increasing trends of micronuclei prevalence with Chornobyl-cleanup-associated dose, and indications of reduction in radiation-associated excess prevalence of micronuclei with time after exposure. There are also indications of substantially increased micronuclei associated with work as an industrial radiographer. This analysis adds to the understanding of the long-term effects of low-dose radiation exposures on relevant cellular structures and methods appropriate for long-term radiation biodosimetry.

**Electronic supplementary material:**

The online version of this article (doi:10.1186/s12940-017-0273-x) contains supplementary material, which is available to authorized users.

## Background

Ionizing radiation is a well-known carcinogen in humans, and a known clastogen leading to broken chromosomes. Chromosomal aberrations [1, 2] and more specifically formation of micronuclei (MN) in cell cytoplasm [3] could represent an early biological predictor of cancer risk. A cytological consequence of the induction of chromosome aberrations is the formation of MN that are observed in interphase cells as a result of a breakdown in repair of chromosomal breaks and general dysfunction of the chromosomal apparatus. MN originate from chromosome fragments or whole chromosomes that are not included in the main daughter nuclei during nuclear division [4–6]. MN rates in peripheral blood lymphocytes (PBL) are moderately rare, typically occurring at levels of 5–25 / 1000 cells [3] and at generally somewhat lower levels in the buccal mucosa, in the range 0.5–10 / 1000 cells [7, 8]. However, despite their moderate rarity, binucleated cells can be easily observed and studied [5, 8]. MN rates generally increase with age [9], although this is not universally observed [10]. MN can also be affected by cigarette smoking [9] and ionizing radiation dose [10], with a complex dependence on radiation dose, radiation energy and dose rate [11–13]. Because cells with MN will not generally pass through mitosis, radiation-induced MN generally decay fairly rapidly (over a period of years) after exposure [14].

It has been suggested that MN are suitable for biomonitoring genetic damage rates [5, 15, 16], in particular damage arising from ionizing radiation exposure, although this is rendered more difficult by the dependence of MN prevalence on age and cigarette smoking. Many studies have shown that the number of radiation-induced MN is strongly correlated with dose and quality of radiation [17–21]. The dose response for MN following a single acute low LET radiation exposure is known to be strongly curvilinear (upward curving), although a more linear relationship is known for high LET radiation exposure [22]. After whole-body exposure with low linear energy transfer (LET) radiation, doses down to 0.1 Gy can be detected [22].

Sari-Minodier et al. [4] evaluated the induction of MN in relation to occupational radiation exposure in a group of 132 exposed hospital workers and 69 controls, adjusting for the possible confounding effects of gender, age, smoking status, familial cancer history and medical irradiation. They demonstrated more frequent MN in the exposed group compared with controls despite the very low levels of exposure (generally <5 mSv / year) [4].

Several studies have been conducted in Chornobyl-exposed children in Belarus, demonstrating elevated rates of MN compared to those from the control areas with little or no radiation exposure [16, 23, 24]. A study of MN in intact cells exposed to serum samples from Chornobyl cleanup workers in Belarus 20 years after exposure observed a significant elevation in MN counts, suggestive of clastogenic factors in their exposed serum which could induce instability [25]. It has been suggested that radiation is one of a number of agents capable of inducing such MN-associated instability [15].

The present study is aimed at evaluating MN in buccal cells in a group of liquidators (cleanup workers) following the Chornobyl accident, collected 16–18 years after exposure. We assess the frequency of MN in relation to various environmental and occupational risk factors, with particular emphasis on assessing excess MN associated with radiation exposure from the Chornobyl accident, and possible modifications by age at exposure and the time between radiation exposure and buccal cell sampling.

## Methods

### Subjects

The study included 111 male members (mean age at first exposure 44.0 years; range 27.8–63.0 years) of the Ukrainian cohort of cleanup workers, who were exposed to different doses of ionizing radiation during the cleanup activities at the Chornobyl nuclear power plant. Subject recruitment and study methods of the previous case-control studies of leukemia nested in the cohort have been described previously [26, 27]. Briefly, all subjects underwent interview for dose reconstruction using the special questionnaire and doses were reconstructed using the Realistic Analytical Dose Reconstruction with Uncertainty Estimation (RADRUE) method [28]. RADRUE has been subject to extensive validation, which we shall consider further in the Discussion. Information about the influence of environmental factors other than Chornobyl radiation, habits, and therapeutic and diagnostic radiological procedures was collected via a special questionnaire [29]. During interview, study participants were asked to donate buccal cell samples. The majority of those who agreed were controls previously involved in case-control studies [26, 27], but a small number of cases (*n* = 3) also agreed to donate buccal cell samples. The National Research Center for Radiation Medicine (NRCRM) in Kyiv and University of California San Francisco (UCSF) Institutional Review Boards approved this study. Before enrollment, each patient gave written informed consent.

### Collection of buccal cell samples for MN analysis.

Exfoliated buccal cells were obtained from the study subjects between November 2002 and March 2004, i.e., 16 to 18 years after the Chornobyl accident. After signing the consent form, commercial “Scope” mouthwash from a plastic container was vigorously swished by the subject in their mouth for 45 s. The throat was not cleared or gargled during the procedure. The mouthwash was then expectorated back into the container which was held close to the mouth. The containers were then tightly sealed and sent back to NRCRM within 24 h of the sample collection. The mouthwash samples were transferred to a 15 ml conical tube and centrifuged at 1500 x g for 15 min. The supernatant was decanted, and the cell pellet was resuspended in 3 ml of TE buffer solution [Tris-EDTA (100 x concentration; pH 8.0) in diethylpyrocarbonate (DEPC) treated water; 1:100 solution]. The 3 ml suspension of cells was distributed into cryovials and stored at -80C. The cells were re-suspended, dropped onto slides and dried at room temperature, and staining solution added, consisting of 10 ml of 0.5% propidium iodide in phosphate-buffered saline (PBS) containing 10% Glycerol. We assess the advantages of this method of buccal cell sampling in comparison with phlebotomy-derived PBL and cytobrush-exfoliated buccal cell samples in the Discussion.

**Fig. 1:**
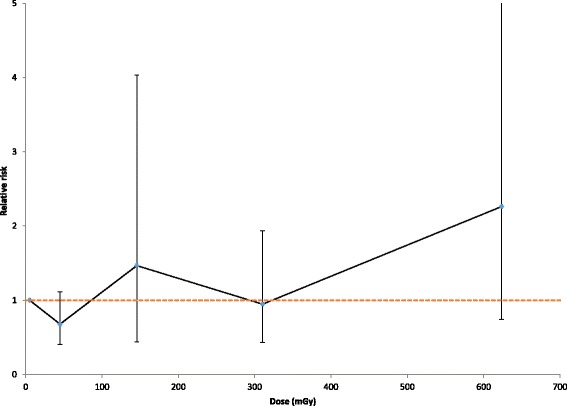
Relative prevalence of micronuclei (MN) in relation to radiation dose, with 95% CI. Background rate adjusted as for models in Tables 2, 3, 4 and 5, and confidence intervals adjusted for overdispersion. 6 individuals with cancer are excluded

Because of a priori doubts as to the effect of prior chemotherapy and radiotherapy, most analyses (Tables 1, 2 and 3, Fig. 1) exclude 6 persons recorded as having a previous tumor, the three persons reporting treatment for radiotherapy or chemotherapy for cancer (one person reporting both types of therapy), and the three leukemia cases known from a previous study [27]. Exclusion of these 6 case resulted in an analysis dataset of 105 persons. However, we also provide in Appendix 2 Tables 8 and 9 certain analyses with these 6 individuals included.

**Table 1:** Influence of various potentially hazardous factors on micronuclei (MN) counts (after exclusion of 6 cancer cases)

		Number	%	Translocation rate		
				Mean MN / 1000 scored	SD	*p*-value heterogeneity
Dose (mGy)	0–19	62	59.0	3.91	5.78	0.4556
	20–99	29	27.6	3.48	4.32	
	100–249	4	3.8	8.50	11.21	
	250–499	7	6.7	4.95	4.11	
	≥500	3	2.9	3.67	0.58	
Age at buccal sample (years)	40–49	19	18.1	4.21	7.08	0.7758
	50–59	32	30.5	3.40	4.98	
	60–69	38	36.2	5.12	5.61	
	≥70	16	15.2	2.48	3.67	
Years since last exposure	< 12	12	10.8	4.00	5.03	0.6956
	12–13	34	30.6	5.04	6.74	
	≥ 14	57	51.4	3.45	4.77	
	Unknown	2	1.8	3.50	4.95	
Days of active work in Chornobyl exclusion zone	0	NA	NA	NA	NA	0.7813
	1–29	19	17.1	3.30	4.40	
	30–59	45	40.5	3.87	5.36	
	60–89	20	18.0	6.03	7.89	
	90–179	14	12.6	3.47	3.53	
	≥ 180	7	6.3	2.48	3.21	
Smoking status	Never smoker/missing	40	38.1	3.85	5.85	0.3187
	Former and current smokers	65	61.9	4.14	5.30	
Alcohol consumption status	Never drinker/missing	24	22.9	3.82	6.50	0.6585
	Former and current drinkers	81	77.1	4.09	5.20	
Work as industrial radiographer	Never	101	96.2	4.03	5.45	0.0516
	Ever	1	1.0	15.00	0.00	
	Unknown	3	2.9	0.33	0.58	
Work with potential radiation exposure (other than Chornobyl)	Never	90	85.7	4.27	5.71	0.5483
	Ever	14	13.3	2.74	3.89	
	Unknown	1	1.0	1.00	0.00	
Work in nuclear industry (including Chornobyl NPP)	Never	97	92.4	4.18	5.67	0.1794
	Ever	5	4.8	3.27	0.83	
	Unknown	3	2.9	0.33	0.58	
Work in army with potential radiation exposure (excluding Chornobyl)	Never	98	93.3	4.28	5.60	0.0307
	Ever	5	4.8	0.60	0.89	
	Unknown	2	1.9	0.50	0.71	
Work with potential radiation exposure (excluding Chornobyl)	Never	100	95.2	4.18	5.57	0.2626
	Ever	3	2.9	1.33	2.31	
	Unknown	2	1.9	0.50	0.71	
Radiotherapy for conditions other than cancer	Never	103	98.1	4.04	5.52	0.3920
	Ever	1	1.0	6.67	0.00	
	Unknown	1	1.0	0.00	0.00	
Any dental X-rays	Never	43	41.0	4.23	6.11	0.1709
	Ever	62	59.0	3.89	5.06	
Any chest X-rays	Never	53	50.5	3.42	5.52	0.4753
	Ever	50	47.6	4.71	5.51	
	Unknown	2	1.9	3.33	4.71	
Any bone X-rays	Never	57	54.3	4.51	6.37	0.3192
	Ever	47	44.8	3.54	4.22	
	Unknown	1	1.0	0.00	0.00	
X-rays other than dental, chest, bone	Never	73	69.5	3.65	5.58	0.0776
	Ever	31	29.5	5.06	5.27	
	Unknown	1	1.0	0.00	0.00	
Total		105	100.0	4.03	5.49	

Heterogeneity *p*-values are adjusted for overdispersion

**Table 2:** Relative risk of various non-Chornobyl radiation-related factors on risk of micronucleus prevalence (after exclusion of 6 cancer cases)

Relative risk category	Relative risk (95% CI)	*p*-value (for heterogeneity unless otherwise stated)
Work as industrial radiographer		
Ever *vs* never + unknown	6.19 (0.90, 31.08)	0.0729
Has subject ever worked with radiation (apart from Chornobyl)		
Ever *vs* never + unknown	1.68 (0.68, 4.03)	0.2584
Has subject ever worked with radiation in the nuclear industry (including nuclear power plant, apart from work in Chornobyl area)		
Ever *vs* never + unknown	1.61 (0.62, 3.98)	0.3195
Has subject ever worked with radiation in the army (apart from work in Chornobyl area)		
Ever *vs* never + unknown	0.27 (0.03, 1.14)	0.0721
Other work with potential radiation exposure		
Ever *vs* never + unknown	1.36 (0.22, 7.29)	0.7283
Radiotherapy for other reasons other than cancer		
Ever *vs* never + unknown	1.73 (0.19, 8.07)	0.5740
Dental X-rays		
Ever *vs* never + unknown	2.94 (0.09, 19.65)	0.4539
per X-ray	0.97 (0.85, 1.11)	0.6917^a^
Chest X-rays		
Ever *vs* never + unknown	1.33 (0.82, 2.17)	0.2505
per X-ray	0.98 (0.90, 1.03)	0.4231^a^
Bone X-rays		
Ever *vs* never + unknown	0.60 (0.37, 0.96)	0.0388
per X-ray	0.92 (0.75, 1.10)	0.3845^a^
X-rays other than dental, chest, bone		
Ever *vs* never + unknown	2.94 (0.09, 19.89)	0.4539
per X-ray	1.15 (0.90, 1.45)	0.2853^a^

All *p*-values relate to improvement in fit of the model with the specific variable added, adjusted for the set of background variables that minimize Akaike Information Criterion (AIC) (as in Appendix 1 Table 7) (with that variable omitted if already included in the optimal background model). Heterogeneity *p*-values and confidence intervals are adjusted for overdispersion

^a^ *p*-value of trend

**Table 3:** Regression analysis of radiation-associated absolute and relative risk of micronucleus prevalence (after exclusion of 6 cancer cases)

Model number	Linear term for absolute risk (*α*) model (/1000 Scored cells /Gy)	Linear term for relative risk (*α*) (/Gy)	Exponential term (*β*) (/Gy)	Deviance (df)	*p*-value
	AIC-minimizing background model (no dose term)				
1	**-**	**-**	**-**	106.04 (75)	
	Absolute risk model in dose (1), background adjusted using AIC-minimizing model				
2	3.03 (−0.78^a^, 7.65)	-		102.56 (74)	0.1170^b^
3	0.71 (−3.05^a^, 16.40)	-	2.51 (−5.36, 11.05^a^)	102.10 (73)	0.5710^c^
	Relative risk model in dose (2), background adjusted using AIC-minimizing model				
4	**-**	1.19 (−0.49, 3.93)	-	103.60 (74)	0.1902^b^
5	**-**	0.14 (−0.97^a^, 7.98)	4.27 (−8.36^a^, 16.89^a^)	102.67 (73)	0.4205^d^

All *p*-values relate improvement in fit evaluated via an *F*-test. All models adjust for background variables that minimize Akaike Information Criterion (AIC), as in Appendix 1 Table 7. 2-sided *p*-values and confidence intervals are adjusted for overdispersion

^a^Wald-based CI

^b^ *p*-value of improvement in fit over null model in dose (model number 1)

^c^ *p*-value of improvement in fit over linear model in dose (model number 2)

^d^ *p*-value of improvement in fit over linear model in dose (model number 4)

### Microscopy

Slides each containing 1000 binucleated cells were scored for micronuclei via oil immersion light microscopy with 900× (40 × 15 × 1.5) magnification. Binucleated cells were scanned and the number of cells with micronuclei was counted. The slides were randomized and coded so that the scorer was not aware of subject ID or dose. The criteria for selecting binucleated cells to score are the following:

a) binucleated cells with main nuclei that are separate and of approximately equal size;

b) main nuclei that touch and even overlap as long as nuclear boundaries can be distinguished; and

c) main nuclei that are linked by nucleoplasmic bridges.

Not scored cells included: trinucleated, quadronucleated, or other multinucleated cells.

### Dosimetry

A time-and-motion method of retrospective dose reconstruction in cleanup workers, known as RADRUE, was developed for the Leukemia Study and for a similar study conducted in Belarus, Russia, and Baltic countries [30] by an international group of scientists including experts from Belarus, France, Russia, the United States, and Ukraine [31, 32]. The method used combined data on work history from dosimetric questionnaires with field radioactivity measurements to estimate individual bone marrow doses for all study subjects. In-person interviews were conducted by trained interviewers and included questions concerning locations of work and residence while in the 30-km exclusion zone around the Chornobyl nuclear power plant, types of work, transportation routes, and corresponding dates. All study subjects were necessarily alive at the time of buccal cell sampling and the associated interview, so that there is not the complication of use of proxy subjects that previous analyses of the underlying case-control dataset have faced [26, 27]. Our analyses were based on the cumulative doses derived as the sums of the arithmetic means of the annual 1986–1990 bone marrow doses estimated by generating 10,000 realizations of dose predictions from RADRUE [31].

### Statistical methods

To evaluate the relationship between cumulative estimated dose and MN, we fitted an additive model, linear-exponential in dose, in which the expected number of MN for individual *i* was given by:

1 $$ {\mu}_i={Scored}_i\left( \exp \left[\sum_{l=1}^N{\gamma}_l{Z}_{i l}\right]+\alpha {D}_i \exp \left[\beta {D}_i\right]\right) $$

where *Scored*_*i*_ is the number of whole scored cells, ${\left({Z}_{il}\right)}_{l=1}^N$are various other variables (including a polynomial function of age at sampling, cigarette smoking, alcohol consumption, chemotherapy and radiotherapy exposure, diagnostic X-rays etc), and *D*_*i*_ is the total buccal dose (in Gy), derived from questionnaire-assessed Chornobyl-related exposure (via RADRUE). The linear-exponential form of dose response used in model (1) is a standard one in analysis of radiobiological data [33], with the linear term *αD*_*i*_ representing the MN induction effect, and the exponential term exp[*βD*_*i*_] representing a sterilization effect. We also fitted a model for the relative MN rate, again linear-exponential in dose:

2 $$ {\mu}_i={Scored}_i \exp \left[\sum_{l=1}^N{\gamma}_l{Z}_{i l}\right]\left(1+\alpha {D}_i \exp \left[\beta {D}_i\right]\right) $$

Exposure to various medical and occupational risk factors was qualitatively assessed, and taken into account in the regression model via the terms ${\left({Z}_{il}\right)}_{l=1}^N$. In order to adequately fit MN prevalence taking account of all factors other than radiation, we considered variables taken from a candidate set of variables that included various occupational (including Chornobyl-related), medical and other terms, some derived from a previous study of these workers [27], and polynomial terms in the (centered) age at buccal sampling, (*age*_*i*_ − 60.104)^*k*^, with integral *k* between 1 and 8, given in Appendix 1 Table 6. Age at buccal sampling, *age*_*i*_, was centered at its mean value, 60.104 years in the full sample, in order to improve the stability of parameter estimates. In order to avoid over-parameterized models, the Akaike Information Criterion (AIC) [34, 35] was employed to select the optimal subset of descriptive variables from this set. AIC penalizes against overfitting by adding 2 x [number of fitted parameters] to the model deviance. A mixed forward-backward stepwise algorithm was used to select the set of variables minimizing AIC, using R [36]. The indicated optimal models were augmented to make them polynomially complete in age at buccal sampling, so that if the optimal model included a variable (*age*_*i*_ − 60.104)^*k*^ for some index *k*, then all terms (*age*_*i*_ − 60.104)^*m*^ for indices 0 ≤ *m* ≤ *k* were also included in the model. The optimal set of variables are given in Appendix 1 Table 7.

Additional models analogous to models (1) and (2) were also fitted, in which adjustment was made for the modifying effects of age at first exposure, *e*_*i*_, or time between last exposure and buccal cell sampling, *t*_*i*_, on the radiation-associated absolute excess risk:

3 $$ {\mu}_i={Scored}_i\left( \exp \left[\sum_{l=1}^N{\gamma}_l{Z}_{i l}\right]+\alpha {D}_i \exp \left[\delta \left({e}_i-43.593\right)+\phi \left({t}_i-15.870\right)\right]\right) $$

and the radiation-associated relative excess risk

4 $$ {\mu}_i={Scored}_i \exp \left[\sum_{l=1}^N{\gamma}_l{Z}_{i l}\right]\left(1+\alpha {D}_i \exp \left[\delta \left({e}_i-43.593\right)+\phi \left({t}_i-15.870\right)\right]\right) $$

The age at first exposure and time since last exposure variables were centered by subtracting their mean values, 43.593 years and 15.870 years among those without cancer, respectively, in order to stabilize parameter estimates. [It should be noted that without a modifying effect of age (or other variables) in the background term, $\exp \left[\sum_{l=1}^N{\gamma}_l{Z}_{il}\right]$, precisely the same *p*-values and estimates of these coefficients (*δ* , *ϕ*) would be obtained in the relative risk model (4) as with the additive model (3), a consequence of the algebraic equivalence of these models in this special case.] Tables 2, 3, 4 and 5 detail the model fits to the MN frequency data, via Poisson maximum likelihood, and associated parameter estimates. Because of indications of marked over-dispersion, in the analyses (Tables 1, 2, 3, 4 and 5) all *p*-values (which are 2-sided) are computed using *F*-tests, derived from quasi-likelihood techniques [37], and the square roots of the associated variance inflation factors (defined by *ϕ* = *deviance*/*df*) are used to scale profile-likelihood confidence intervals in the standard way, i.e., by multiplying the distance of confidence limit to the best estimate by the square root of the inflation factor. Very similar estimates of the variance inflation factor derived in this way, which after adjustment for the background variables were about 1.4–1.5, were yielded by use of quasi-likelihood models in R [36], taking account of overdispersion [37]. Models were fitted using R [36] and Epicure [38]. Although all *p*-values are 2-sided, arguably 1-sided *p*-values (which would be about half the values given) are possibly more relevant for certain of the tests that we perform, given that one would expect MN prevalence only to increase with increasing dose. We emphasize in a few relevant places the two-sided nature of the relevant tests.

**Table 4:** Regression analysis of modifying effect of age at first exposure and time since last exposure on absolute and relative radiation-associated excess risk of micronucleus prevalence (after exclusion of 6 cancer cases)

	Age at first exposure (*δ*)(year^−1^)	Time since last exposure (ϕ)(year^−1^)
Absolute risk model (1)		
Coefficient	−0.088 (−0.294^a^, 0.079)	−0.464 (−1.249, 0.371)
*p*-value	0.3215	0.2733
Relative risk model (2)		
Coefficient	−0.237 (−1.055^a^, 0.069)	−0.753 (−23.843, 0.118)
*p*-value	0.1368	0.0949

All *p*-values relate improvement in fit of the model to a model without adjustment for the specific temporal effect (age at first exposure and time since last exposure), using models of the form considered in Tables 2 and 3, evaluated via an *F*-test. All models adjust for background variables that minimize Akaike Information Criterion (AIC), as in Appendix 1 Table 7. *p*-values and confidence intervals are adjusted for overdispersion

^a^Wald-based CI

**Table 5:** Regression analysis of modifying effect on radiation dose of days (active) work in the Chornobyl exclusion zone, liquidator type, cigarette smoking and alcohol consumption on absolute and relative excess risk of micronucleus prevalence data (after exclusion of 6 cancer cases)

Modifying effect	*p*-value	
	Absolute risk model (1)	Relative risk model (2)
Days work in Chornobyl exclusion zone^a^	0.2808	0.1671
Days active work in Chornobyl exclusion zone^b^	0.2590	0.2156
Liquidator type^c^	0.6681	0.7678
Cigarette smoking^d^	0.8232	0.6922
Alcohol consumption^e^	0.7291	0.7246

All *p*-values relate improvement in fit of a linear model in radiation dose without adjustment to the radiation term for the specific effect (days (active) work in Chornobyl exclusion zone, liquidator type, smoking consumption, alcohol consumption), using models of the form considered in Tables 2, 3 and 4, evaluated via an *F*-test. All models adjust for background variables that minimize Akaike Information Criterion (AIC), as in Appendix 1 Table 7. *p*-values are adjusted for overdispersion

^a^based on days work in Chornobyl exclusion zone treated as a categorical variable (unknown number of days or number < 30, days work ≥30 and <90, days work ≥90)

^b^based on days active work in Chornobyl exclusion zone treated as a categorical variable (number of active days <30, active days work ≥30 and <90, active days work ≥90)

^c^based on liquidator work treated as a categorical variable (unknown + early responders + firefighters + nuclear power workers, military personnel, drivers + construction workers)

^d^based on cigarette smoking treated as a categorical variable (unknown smoking status or never smoker, 1–9 cigarettes per day, ≥10 cigarettes per day)

^e^ based on alcohol consumption treated as a categorical variable (never + drinks once per month or less, drinks 2–3 times per month + once per week, drinks more than once per week + every day)

## Results

Among the 105 persons without previous cancer the estimated mean Chornobyl-related bone marrow dose was 59.5 mSv (range 0–748.4 mSv) and the mean age at first exposure was 43.6 years (range 27.8–63.0 years). Among the same group with information on years since last exposure, the mean years since last exposure was 15.9 years (range 12.4–17.8 years). There are variations of MN prevalence with days of active work in the Chornobyl exclusion zone, being particularly high among those working 50–99 days, by type of work as a cleanup worker, and among those 25 persons in military occupations (Table 1). Minimizing AIC led to inclusion of 11 variables, providing a parsimonious model of underlying MN prevalence that includes a 5th order polynomial in age at buccal sample (Appendix 1 Table 7).

Table 2 demonstrates that there is a borderline significant increase in MN frequency among those reporting work as an industrial radiographer, with a relative risk of 6.19 (95% CI 0.90, 31.08, *p* = 0.0729). There are weak indications of increased MN frequency associated with radiation work apart from Chornobyl, or with radiation work in the nuclear industry, with relative risks of 1.68 (95% CI 0.68, 4.03, *p* = 0.2584), and 1.61 (95% CI 0.62, 3.98, *p* = 0.3195), respectively (Table 2), although there was a borderline significant reduction in MN prevalence among those reporting radiation work in the army, with a relative risk of 0.27 (95% CI 0.03, 1.14, *p* = 0.0721) (Table 2). There was a significant decrease in numbers of MN among those reporting bone X-rays, with a relative risk of 0.60 (95% CI 0.37, 0.96, *p* = 0.0388), although there were only weak indications of a (negative) trend with numbers of bone X-rays (*p* = 0.3845) (Table 2).

Table 3 demonstrates that after adjustment for some of these variables, there are borderline significant indications of a positive Chornobyl-related radiation dose response for the absolute risk of MN of 3.03 MN per 1000 scored cells per Gy (95% CI -0.78, 7.65, 2-sided *p* = 0.1170); there is somewhat weaker evidence of such trends using a relative risk model (2-sided *p* = 0.1902). There was some elevation of MN in a group exposed to intermediate levels of dose, 100–250 mGy, also at higher levels of dose, 500+ mGy (Fig. 1, Table 1). There is no evidence of a linear-exponential dose response, whether using absolute or relative risk models (*p* = 0.5710, *p* = 0.4205, respectively, Table 3).

Table 4 demonstrates that there was a borderline significant reduction of excess relative MN prevalence with increasing time since last exposure (*p* = 0.0949), and there are somewhat weaker indications (*p* = 0.2733) of a reducing trend in absolute excess MN prevalence with increasing time since last exposure. Variations of radiation-associated MN prevalence with age at first exposure are somewhat weaker, whether using relative risk or absolute risk models (*p* = 0.1368, *p* = 0.3215, respectively). The modifying effect of time since last exposure, whether on relative or absolute risk, is notably large, *ϕ* =  − 0.753 year^−1^ or *ϕ* =  − 0.464 year^−1^, respectively. Table 5 demonstrates that there are only weak indications of significant modifying effects on the MN Chornobyl-related dose response by number of days of work, or by number of active days of work, in the Chornobyl exclusion zone, whether in relation to absolute (*p* = 0.2808, *p* = 0.2590, respectively) or relative risk (*p* = 0.1671, *p* = 0.2156, respectively) models. There are no indications of modifications of MN prevalence by type of liquidator, cigarette smoking, or alcohol consumption, whether in relation to absolute (*p* = 0.6681, *p* = 0.8232, *p* = 0.7291, respectively) or relative risk (*p* = 0.7678, *p* = 0.6922, *p* = 0.7246, respectively) (Table 5).

Analyses including the 6 individuals with cancer were not markedly different (Appendix 2 Tables 8 and 9).

## Discussion

We observed a large and borderline significant (2-sided *p* = 0.0729) increase in MN frequency among those reporting work as an industrial radiographer, although this finding was based on a single case. We also observed borderline-significant indications of a positive Chornobyl-related radiation dose response for MN (2-sided *p* = 0.1170). There is a substantial and borderline significant (*p* = 0.0949) reduction of Chornobyl-dose-related relative prevalence with increasing time since last exposure. We observed a significant decrease in numbers of MN among those reporting bone X-rays (2-sided *p* = 0.0388), but there was no significant trend in MN prevalence with numbers of bone X-rays.

The MN assay using buccal cells is a minimally invasive method for studying DNA damage, chromosomal instability, cell death and the regenerative potential of human buccal mucosal tissue. This method is increasingly used in molecular epidemiological studies for investigating the impact of nutrition, lifestyle factors, genotoxic exposure and genotype on DNA damage, chromosome mis-segregation and cell death. Although MN in buccal mucosa occur at a lower frequency than in PBL [3, 7, 8], there is no reason to exclude their use for this reason. Biologically one would expect the excess MN to persist to the same relative extent in various tissues of the body, so that it is not unreasonable to examine any radiation-exposed tissue in the body, in particular the buccal mucosa, for this marker of exposure. Although there are some very high skin doses due to low energy beta emitters [39], in the Chornobyl liquidators “photon energy spectra varied from one location to another and from one time period to another but were generally centered between 0.15 and 0.4 MeV” [32]. In the range of energy from 0.15 to 0.4 MeV, dose does not vary much with energy or from one organ/tissue to another [40]. Both blood and epithelial tissue are highly regenerative tissues with a continuous need for repopulation and a base of stem cells needed for lifetime renewal. Because of the long time between exposure and sampling, the damage measured here is primarily of the buccal stem cells. The biomarkers measured in this assay have been associated with increased risk of accelerated ageing, cancer and neurodegenerative diseases [41]. However, because cells with MN will not generally pass through mitosis, MN generally decay fairly rapidly, over a few years, after mutagenic exposure. In particular, there is human in vivo data demonstrating this. A study of a pregnant woman and her then *in utero* child, both heavily exposed (to several Gy) from an orphan ^60^Co source, demonstrated a progressive decline in MN in mother and daughter over the period from 41 days to 16 years after the accident [42]. This may also explain our generally null findings in relation to radiation exposure from the Chornobyl accident, last exposure from which occurred between 12 and 18 years prior to buccal cell sampling. Although not statistically significant, there is a pronounced negative modifying effect of time since exposure on the association of MN with radiation dose (Table 4).

The excess of MN that we observed among those reporting work as industrial radiographers, with a relative risk of 6.19 (95% CI 0.90, 31.08) (Table 2), should be compared with those in a study of Sari-Minodier et al. [43], who found a smaller, approximately two-fold, increase in a group of 29 radiographers compared with a group of 24 controls. The confidence intervals on our relative risks are wide, and so the relative risks in these two groups are statistically compatible despite these disparate point estimates.

Increases in MN frequency have been observed in some groups exposed to dental and other diagnostic X-rays procedures [7, 44], although not in many others [45–49]. In all these studies comparisons were made of MN within subjects before and shortly after (generally within a few days) of X-ray exposure. It is interesting that although negative for MN, the studies of Ribeiro et al. [45, 47], Angelieri et al. [46, 49] and Lorenzoni et al. [48] showed increases in the cytotoxic endpoints of karrhyorexis, pyknosis, and karyolysis after X-ray exposure, suggesting a substantial degree of induced cell death among buccal mucosa. This may explain the reduced MN frequency that we observed, presumably a much longer time after bone X-ray exposure than in these earlier studies. Unfortunately the cytotoxic endpoints considered in these earlier studies (karrhyorexis, pyknosis, and karyolysis) were not evaluated here. In addition to induced cell death of damaged mucosa, bone X-ray exposure may stimulate enhanced DNA repair as a hormetic effect [50].

A study of 132 radiation-exposed hospital workers and 69 controls matched for gender, age and smoking habits previously showed that chromosomal damage leading to micronucleated lymphocytes is more frequent after exposure to ionizing radiation than in controls, despite the very low dose levels recorded during a 10-year period [4]. The effect was significantly greater in females than in males, and a significant correlation between age and MN rates was observed in females but not in males. There was no significant effect of cigarette smoking. A large study of 1392 radiation workers and 143 controls in China demonstrated increased MN prevalence at relatively low occupational radiation doses (~50 mSv equivalent dose), and variation of MN prevalence also with exposure time [51]. A significant excess prevalence (*p* < 0.001) of MN in PBL was observed in a group of 25 exposed male workers compared with a group of 25 age-matched controls, with borderline significant indications (*p* = 0.079) of an increasing dose response [52]; exposure occurred 32–41 years previously. In PBL of patients receiving radiotherapy, MN yield increased with increasing equivalent dose and there was a general decline in MN yields with increasing length of follow-up, with considerable variation between individuals [53]. Whether this variation reflects inter-individual differences in susceptibility to radiation-induced DNA damage, or simply inter-individual differences in ability to clear MN, is unknown. However, in the period from 19 to 75 months after treatment, seven of thirteen patients showed higher MN yields than their respective levels before radiotherapy, indicating the persistence of radiation-induced residual cytogenetic damage in some subjects [53]. Such differences could be influenced by numerous subject characteristics, including germline genetics. However, the relatively short interval between radiation exposure and sampling should be noted. Because we performed analysis a long time after radiation exposure (e.g., at least 12.4 years after exposure from the Chornobyl accident) we used various methods to attempt to increase the MN assay sensitivity, although we did not use the cytokinesis-block micronucleus test (CB-MNT) [54]. The main analysis assessed counts of binucleated cells, commonly regarded as a type of cell with defective cytokinesis [8].

The method of alcohol-based mouthwash-expectorated buccal cell sampling that we employ is less commonly used than the cytobrush-exfoliated buccal cell collection method [9]. However, there have been a number of recent studies in the literature that use this method of mouthwash-mediated buccal cell collection, in particular those of Lum and Le Marchand [55], Garcia-Closas et al. [56], and Andrisin et al. [57]. As highlighted by Garcia-Closas et al. [56] and Lum and Le Marchand [55] the use of alcohol-based mouthwash has certain decisive advantages over cytobrush-exfoliated buccal cell collection in the proportion of high molecular weight DNA extracted and its stability, and the much lower proportion of non-human (bacterial) DNA content. As shown by Andrisin et al. [57] the method also produces samples with stable DNA content, that can be kept at room temperature for up to 90 days, unlike cytobrush-exfoliated buccal cell samples, that require processing within a matter of days; as shown by Walsh et al. [58] there is significant bacterial contamination on cytobrush-collected buccal samples within 4 days of sample collection. The method is also more acceptable to study subjects than PBL samples obtained via phlebotomy or cytobrush-exfoliated buccal cell collection, being less invasive; it is also preferable to saline-mouthwash collected buccal samples because of the more agreeable aftertaste. Admittedly not all of these advantages of the mouthwash method matter here, since all samples were processed within a few days of collection. Also, as discussed in the Introduction the baseline frequency of MN produced by this method is lower than for PBL.

A refinement of the assay, using a pan-centromeric DNA probe labeling the centromeric region, discriminates between centromere-negative MN (C-MN) and centromere-positive MN (C+MN). Significantly higher frequency of C-MN have been observed using this assay in a number of radiation-exposed groups exposed at relatively low levels of radiation dose (generally <100 mSv) [43, 59], also in an individual receiving a large (65 Gy) therapeutic dose [60]. The finding of significantly elevated MN prevalence in immortalized HPV-G cells exposed to serum samples from Chornobyl cleanup workers from Belarus some 20 years after exposure is of interest [25]. However, the study did not directly examine the rates of MN in the blood cells of the Belarus cleanup workers, but rather the effects of the soluble factors contained in sera from radiation-exposed subjects on human immortalized reporter cells treated with these sera, and as such is only tangentially relevant to the present study.

Set against that, there are a number of null studies of MN in buccal cells. A study of 15 heavy smokers and 17 non-smokers exposed to panoramic dental X-rays evaluated MN in exfoliated oral mucosa cells, and found no statistically significant differences in MN rates before versus after exposure either in smokers or non-smokers, although there were differences in measures of induced cell death (pyknosis, karyolysis, and karyorrhexis) [46]. A study of 31 healthy individuals evaluated MN before and 10 days after X-ray dental radiography and observed similar frequencies of MN, karyolysis and pyknosis over time (*p* > 0.90), although chromatin condensation and karyorrhexis increased significantly after exposure (*p* < 0.0001) [61]. These and other studies suggest an absence of MN induction after low dose radiation exposure, although there appear to be cytotoxic effects caused by increasing apoptosis [61, 62].

There has also been no demonstrated dose-related increase in MN frequency in the bone marrow cells of a small sample of Hiroshima atomic bomb survivors [63]. It is possible that the interval between exposure and sampling, about 5 years, may have contributed to this null finding. Interestingly, Oesterle and Finch observed internuclear bridges in the marrow smears in a large proportion of the heavily exposed (>3 Gy) survivors, and a substantial excess karyomere frequency in a group exposed to even larger doses (> 6 Gy) [63].

Bone marrow doses were estimated here by the RADRUE method [28]. RADRUE has been subject to extensive validation, in particular with thermoluminescent dosimetry (TLD) badges worn by certain reliably film-badged groups of workers (AC-605 liquidators), and various biological dosimeters, in particular electron paramagnetic resonance (EPR) in teeth and fluorescence in situ hybridization (FISH) in peripheral blood lymphocytes, as documented in the report of Kryuchkov et al. [32]. Although RADRUE dose estimates would not be biased, nevertheless there are considerable uncertainties in RADRUE doses, which have been estimated to have mean geometric standard deviation (GSD) about 1.9; the GSD was considerably higher, 4–6, when the subject was deceased and a proxy had to be used, but this is not relevant here, because all study subjects were alive at the time of interview [32]. These errors are of complex form, very likely a mixture of Berkson and classical type [64]. Classical dose errors would be expected to bias trends with dose towards the null; Berkson-type errors would not bias dose response trends, but would inflate the associated confidence intervals [64, 65].

Genome sequencing has uncovered a new mutational phenomenon called chromothripsis, characterized by extensive genomic rearrangements and an oscillating pattern of DNA copy number levels, all restricted to a few chromosomes. Using a combination of live-cell imaging and single-cell genome sequencing, Zhang et al. [66] demonstrated that MN formation can generate a spectrum of genomic rearrangements, some of which recapitulate known features of chromothripsis. These events are restricted to the mis-segregated chromosome and occur within one cell division. Zhang et al. [66] demonstrated that the mechanism for chromothripsis may involve the fragmentation and subsequent reassembly of a single chromatid from a MN. Chromothripsis has been linked with proton beam irradiation in vitro [67] and has also been shown to drive telomerase reactivation in CLL [68], the most common cancer experienced by Chornobyl cleanup workers.

In our study buccal cells rather than PBL have been used, but as above the only complication this is likely to introduce is the somewhat lower prevalence of MN. The study has some limitations. The dose from Chornobyl-related exposure was derived from questionnaire-based assessments, using RADRUE, and all other information relating to occupational and medical exposure was also questionnaire-derived. There was no assessment of radiation dose from radiotherapy or from other sources, nor was there any assessment made of the timing of the medical procedures and types of occupational exposure. The design of the questionnaire given to the study subjects made it impossible to determine the sequence of exposure of ionizing radiation and the possible confounding factors. That said, since we found no effect of any other occupational or medical factor, it is perhaps unlikely that there would be confounding due to these factors. Fourteen subjects had evidence of treatment for cancer, but exclusion of these made no difference to inference on dose response. This study gives no answer to the question of whether previous low-dose radiation exposure could change DNA susceptibility to chemo- or radiotherapy and elongate the time of damage persistence, that might be revealed by the MN assay.

As well as the long time interval between Chornobyl-related radiation exposure and buccal cell sampling, another factor that must be considered is the generally modest levels of radiation dose. The mean dose in the present study was 59.5 mSv, with a maximum dose of 748.4 mSv. Data from an experiment involving ex vivo ^60^Co-gamma irradiation of fibroblasts suggests that the minimal dose at which an elevated level of MN can be observed is about 200–250 mGy, minimally dependent on age [69]; only 10 of our cases had doses in excess of 250 mSv (Table 1, Appendix 1 Table 6). Set against that, a number of occupationally radiation-exposed groups observed increases in MN at levels of dose somewhat lower than the present study [4, 51]. A more detailed investigation of our results is still needed. Quantification of the precise dose-response relationship between MN levels and radiation dose in in vitro models could inform future studies in humans. However, to be most germane, such studies must also assess the effects of various time intervals between radiation exposure and cell sampling.

## Conclusions

There are indications of increasing trends of MN prevalence with Chornobyl-cleanup-associated dose, and indications of reduction in radiation-associated excess prevalence of MN with time after radiation exposure. There are also indications of substantially increased MN prevalence associated with work as an industrial radiographer, although based on a single case. This analysis adds to the understanding of the long-term effects of low-dose radiation exposures on relevant cellular structures and methods appropriate for long-term radiation biodosimetry.

## Appendix 1

**Table 6:** Candidate variables used for minimizing Akaike Information Criterion (AIC), via step-AIC algorithm

Variable	Description
first	period of first mission to the Chornobyl zone
days_act	duration of active work in the Chornobyl zone in days
Case_leuk	case of leukemia in the study of Zablotska et al. 2013
Year_first_exposed	year first exposed via work in Chornobyl exclusion zone (calculated from dose record)
Year_last_exposed	year last exposed via work in Chornobyl exclusion zone (calculated from dose record)
proxy^a^	proxy interviews
proxy_str	proxy status based on the cases status in the set
number1	number of missions
first_year	year first exposed via work in Chornobyl exclusion zone
duration	total duration of work in the Chornobyl zone in months
duration_act	duration of active work in the Chornobyl zone in months
days	total duration of work in the Chornobyl zone in days
tse	time since first exposure in the zone until diagnosis (for cases) and reference time (for controls) in years; tse < 0 (*n* = 4) is set to 0
radiation	non-Chornobyl work with radiation exposure
industry	work in hazardous industries
oil	oil or chemical industry
chemical	non-Chornobyl work with hazardous chemicals
petroleum	work in petroleum industry
pesticides	work with pesticides
solvents	work with solvents
other_chemical	work with hazardous chemicals other than in petroleum industry/pesticides/solvents
age	age at diagnosis (for cases) and age at reference date (for controls)
age_x	age at first exposure in the zone
subtype^a^	subtype of leukemia
subtypecll^a^	CLL subtype
subtypecll_str	CLL subtype, based on the case status, matching controls set to case status
y_dx^a^	year of diagnosis; for controls set to −1
reference	reference year set to year of diagnosis (for cases) and for controls is the same as for matched case
y_int	year of interview
liqtype	type of work performed in the Chornobyl zone during the first mission
flag1	additional controls re-distributed among cases
flag2	controls used in risk analysis for Phase I but now matched to their original case used in set Phase II
flag_definite	certainty in the leukemia diagnosis (definite + probable/other)
Phase	study phase (phase I/phase II)
COM1_EXP1	dosimetry expert’s opinion about the quality of interview during the 1st mission to the 30-km exclusion zone
COM2_EXP1	dosimetry expert’s opinion about the quality of interview during the 2nd mission to the 30-km exclusion zone
COM3_EXP1	dosimetry expert’s opinion about the quality of interview during the 3rd mission to the 30-km exclusion zone
QUES1	interviewer’s opinion on the quality of questionnaire: Did subject reply with interest?
QUES2	interviewer’s opinion on the quality of questionnaire: Did the worker remember his job in the 30-km exclusion zone?
Liquid	clean-up worker with data on interviewer’s opinion about the quality of interview
Exp	expert dosimetrist who prepared dosimetric questionnaire for input into RADRUE
time_int_ch	time from interview to chemotherapy (date of start of chemotherapy for CLL and date of diagnosis for non-CLL)
set^a^	cases with direct interviews
t11^a^	if time_int_ch ≠ 99 and time_int_ch ≠ −1 and cases = 1 and proxy = 0 and −1 ≤ time_int_ch ≤ 1 then t11 = 1;else t11 = 0;
t1515^a^	if time_int_ch ≠ 99 and time_int_ch ≠ −1 and cases = 1 and proxy = 0 and −1.5 ≤ time_int_ch ≤ 1.5 then t1515 = 1;else t1515 = 0;
t12^a^	if time_int_ch ≠ 99 and time_int_ch ≠ −1 and cases = 1 and proxy = 0 and −1.0 ≤ time_int_ch ≤ 2.0 then t12 = 1;else t12 = 0;
t22	if time_int_ch ≠ 99 and time_int_ch ≠ −1 and cases = 1 and proxy = 0 and −2.0 < time_int_ch < 2.0 then t22 = 1; else t22 = 0
liqtype_recode	type of work performed in the Chornobyl zone during the first mission recoded (from liqtype) (missing/early responders & firefighters/professional nuclear power workers/military personnel/drivers & construction workers)
num_miss	number of missions to the Chornobyl exclusion zone
rad_exp	study subject ever worked with radiation (other than the time the study subject spent in the Chornobyl area)
rad_radiography	study subject ever worked with radiation as industrial radiographer (other than time study subject spent in the Chornobyl area)
rad_nuclear	study subject ever worked with radiation in the nuclear industry (including NPP, other than time the study subject spent in the Chornobyl area)
rad_army	study subject ever worked with radiation in army service (other than time the study subject spent in the Chornobyl area)
rad_other	study subject ever worked with radiation other than in army/nuclear industry/industrial radiography (other than time the study subject spent in the Chornobyl area)
haz_exp	study subject ever worked in a hazardous industry (specific types of chemical/electrical/road-building/other)
haz_chem	study subject worked in chemical industry/oil industry (hazardous industry)
haz_el	study subject worked in electrical industry (hazardous industry)
haz_road	study subject worked in road-building industry (hazardous industry)
haz_other	study subject worked in one of coal mining/military service/metallurgical industry/welding/gas equipment/other hazardous industries (hazardous industry)
chem_exp	Has the study subject ever worked with hazardous chemicals?
chem_petrol	study subject worked with petrol (petrol and agricultural pesticides/petrol and organic solvents)
chem_pest	study subject worked with agricultural pesticides (petrol and agricultural pesticides/agricultural pesticides and organic solvents/agricultural pesticides and other)
chem_solv^a^	study subject worked with organic solvents (petrol and organic solvents/agricultural pesticides and organic solvents)
chem_other	study subject worked with hazardous chemical other than petroleum/agricultural pesticides/organic solvents
Oblast	Dnipropetrovsk/Kyiv oblast/Kharkiv/Cherkassy/Chernihiv/Kyiv-city
Educ	level of education of interviewee (8 years or less school/high school/trade school/higher education)
Urban	Type of settlement (city/village/other)
radtx^a^	Has the study subject had radiotherapy for cancer treatment
chemotx^a^	Has study subject had chemotherapy for cancer treatment
radtx_other	Has the study subject ever received radiotherapy for medical conditions other than cancer
x_dental	Has study subject ever had dental X-ray?
x_dental_n	Number of times study subject has had dental X-ray
x_bone	Has interviewee ever had bone X-ray?
x_bone_n	Number of times interviewee has had bone X-ray?
x_chest	Has study subject ever had chest X-ray (except fluoroscopy)
x_chest_n	Number of times study subject ever had chest X-ray (except fluoroscopy)
x_other	Has the interviewee ever had other X-ray examinations (than bone, chest, dental)?
x_other_n	How many times interviewee had any other type (than bone, chest, dental) of X-ray examination?
fam_cancer	Have any of interviewee’s relatives had any solid cancer or leukemia (mother or father, or other relative)?

^a^variable excluded from step-AIC analysis when omitting 6 cancer cases because trivial in reduced dataset

**Table 7:** Optimal variable set, minimizing Akaike Information Criterion (AIC), fitted to data excluding any cancers (radtx ≠ 1 AND chemotx ≠ 1 AND Case_leuk = 0)(6 persons excluded) or in complete database. In either case variables are shown in order of selection into the final optimal model

Variable	Description
All cases	
rad_radiography	study subject ever worked with radiation as industrial radiographer (other than time study subject spent in the Chornobyl area)
x_other	Has the interviewee ever had other (than bone, chest, dental) X-ray examination?
chemotx	Has study subject had chemotherapy for cancer tumour
flag2	controls used in risk analysis for Phase I but now matched to their original case used in set Phase II
COM1_EXP1	dosimetry expert’s opinion about the quality of interview during the 1st mission to the 30-km zone
[age at buccal sampling]^7^	polynomial completed to include all lower order terms - [age at buccal sampling]^*k*^ with *k* < 7
exp	expert dosimetrist who prepared dosimetric questionnaire for input into RADRUE
y_int	year of interview
rad_army	study subject ever worked with radiation in army service (other than time the study subject spent in the Chornobyl area)
haz_exp	study subject ever worked in a hazardous industry (specific types of chemical/electrical/road-building/other)
haz_el	study subject worked in electrical industry (hazardous industry)
x_dental_n	number of times study subject had dental X-ray
num_miss	number of missions to the Chornobyl exclusion zone
haz_road	study subject ever worked in road-building industry (hazardous industry)
sm_pday	How many cigarettes did interviewee smoke in a typical day?
x_bone	Has interviewee ever had bone X-ray?
Excluding 6 cancer cases	
x_other_n	How many times interviewee had any other type (than bone, chest, dental) of X-ray examination?
rad_army	study subject ever worked with radiation in army service (other than time the study subject spent in the Chornobyl area)
COM1_EXP1	dosimetry expert’s opinion about the quality of interview during the 1st mission to the 30-km zone
[age at buccal sampling]^5^	polynomial completed to include all lower order terms - [age at buccal sampling]^*k*^ with *k* < 5
exp	expert dosimetrist who prepared dosimetric questionnaire for input into RADRUE
x_dental_n	Number of times study subject had dental X-ray
x_bone	Has interviewee ever had bone X-ray?
industry	work in hazardous industries
sm_pday	How many cigarettes did interviewee smoke in a typical day?
num_miss	number of missions to the Chornobyl exclusion zone
haz_road	study subject ever worked in road-building industry (hazardous industry)
rad_radiography	study subject ever worked with radiation as industrial radiographer (other than time study subject spent in the Chornobyl area)
other_chemical	work with hazardous chemicals other than in petroleum industry/pesticides/solvents
flag_definite	certainty in the leukemia diagnosis (definite + probable/other)
haz_chem	Study subject worked in chemical industry/oil industry (hazardous industry)

## Appendix 2

**Table 8:** Influence of various potentially hazardous factors on micronuclei (MN) counts, using all samples (including 6 cancer cases)

		Number	%	Translocation rate		
				Mean MN / 1000 scored	SD	*p*-value heterogeneity
Dose (mSv)	0–19	66	59.5	4.17	5.84	0.5867
	20–99	29	26.1	3.48	4.32	
	100–249	5	4.5	7.40	10.02	
	250–499	8	7.2	4.71	3.86	
	≥500	3	2.7	3.67	0.58	
Age at buccal sample (years)	40–49	19	17.1	4.21	7.08	0.7879
	50–59	33	29.7	3.37	4.90	
	60–69	40	36.0	5.34	5.74	
	≥70	19	17.1	2.99	3.77	
Years since last exposure	< 12	14	12.6	3.81	4.66	0.4611
	12–13	35	31.5	5.36	6.89	
	≥ 14	59	53.2	3.55	4.77	
	Unknown	3	2.7	3.67	3.51	
Days of active work in Chornobyl exclusion zone	0	1	0.9	4.00	0.00	0.9364
	1–29	21	18.9	4.22	5.18	
	30–59	46	41.4	3.85	5.30	
	60–89	20	18.0	6.03	7.89	
	90–179	15	13.5	3.39	3.41	
	≥ 180	8	7.2	2.54	2.98	
Smoking status	Never smoker/missing	40	36.0	3.85	5.85	0.2428
	Former and current smokers	71	64.0	4.33	5.31	
Alcohol consumption status	Never drinker/missing	25	22.5	3.82	6.36	0.6316
	Former and current drinkers	86	77.5	4.26	5.25	
Work as industrial radiographer	Never	107	96.4	4.16	5.45	0.0475
	Ever	1	0.9	15.00	0.00	
	Unknown	3	2.7	0.33	0.58	
Work with potential radiation exposure (other than Chornobyl)	Never	94	84.7	4.30	5.62	0.6426
	Ever	16	14.4	3.54	4.92	
	Unknown	1	0.9	1.00	0.00	
Work in nuclear industry (including Chornobyl NPP)	Never	102	91.9	4.33	5.68	0.1705
	Ever	6	5.4	3.11	0.84	
	Unknown	3	2.7	0.33	0.58	
Work in army with potential radiation exposure (excluding Chornobyl)	Never	103	92.8	4.29	5.49	0.2015
	Ever	6	5.4	3.17	6.34	
	Unknown	2	1.8	0.50	0.71	
Work with potential radiation exposure (excluding Chornobyl)	Never	106	95.5	4.31	5.56	0.2436
	Ever	3	2.7	1.33	2.31	
	Unknown	2	1.8	0.50	0.71	
Radiotherapy for conditions other than cancer	Never	109	98.2	4.17	5.52	0.3851
	Ever	1	0.9	6.67	0.00	
	Unknown	1	0.9	0.00	0.00	
Any dental X-rays	Never	46	41.4	4.45	6.16	0.1184
	Ever	65	58.6	3.95	5.00	
Any chest X-rays	Never	54	48.6	3.40	5.47	0.1464
	Ever	55	49.5	4.93	5.52	
	Unknown	2	1.8	3.33	4.71	
Any bone X-rays	Never	62	55.9	4.70	6.33	0.2739
	Ever	48	43.2	3.55	4.17	
	Unknown	1	0.9	0.00	0.00	
X-rays other than dental, chest, bone	Never	77	69.4	3.72	5.48	0.0629
	Ever	33	29.7	5.31	5.47	
	Unknown	1	0.9	0.00	0.00	
**Total**		**111**	**100.0**	**4.16**	**5.49**	

Heterogeneity *p*-values are adjusted for overdispersion

**Table 9:** Regression analysis of absolute and relative risk of micronucleus prevalence data in relation to radiation dose, using all samples

Model number	Linear term for absolute risk (*α*) model (/1000 Scored cells /Gy)	Linear term for relative risk (*α*) (/Gy)	Exponential term (*β*) (/Gy)	Deviance (df)	*p*-value
	AIC-minimizing background model (no dose term)				
1	**-**	**-**	**-**	114.05 (80)	
	Absolute risk model in dose (1), background adjusted using AIC-minimizing model				
2	2.90 (−1.26^a^, 7.66)	**-**	**-**	110.90 (79)	0.1379^b^
3	0.28 (−2.28^a^, 21.56)	**-**	3.64 (−9.59^a^, 16.87^a^)	110.73 (78)	0.7310^c^
	Relative risk model in dose (2), background adjusted using AIC-minimizing model				
4	**-**	0.94 (−0.66, 3.68)	**-**	112.50 (79)	0.3003^b^
5	**-**	0.06 (−0.74^a^, 0.86^a^)	4.57 (−16.45^a^, 25.60^a^)	111.72 (78)	0.4625^d^

All *p*-values relate improvement in fit evaluated via an *F*-test

^a^Wald-based CI

^b^ *p*-value of improvement in fit over null model in dose (model number 1)

^c^ *p*-value of improvement in fit over linear model in dose (model number 2)

^d^ *p*-value of improvement in fit over linear model in dose (model number 4)

## Additional file

Additional file 1: Data files (Excel, text) and other R and Epicure files used in the analysis. (ZIP 3032 kb)
